# The first total robotic-assisted lymphovenous anastomosis: prospects for innovation and future development

**DOI:** 10.1007/s11548-026-03589-0

**Published:** 2026-03-04

**Authors:** Carlotta Imholz, Carmen E. Zurfluh, Jennifer A. Watson, Nicole Lindenblatt

**Affiliations:** 1 Department of Plastic Surgery and Hand Surgery, University Hospital Zurich, Rämistrasse 100, 8091 Zurich, Switzerland; 2 University of Zurich, Zurich, Switzerland

**Keywords:** Robotic-assisted, Robotic, Symani, Supermicrosurgery, Lymphovenous anastomosis, LVA, Lymphedema, Robotics

## Abstract

**Purpose:**

Lymphovenous anastomosis is a well-established treatment method for peripheral lymphedema. To date, microsurgical robotic platforms have been used exclusively for the anastomosis of vessels in lymphatic surgery. This study reports on the first total robotic-assisted lymphovenous anastomosis and further highlights future directions for microsurgical robotic assistance in lymphatic surgery.

**Methods:**

Using the Symani® Surgical System, two patients with lower extremity lymphedema received total robotic-assisted lymphovenous anastomoses. This included robotic-assisted microdissection of lymphatic and venous vessels followed by performance of a robotic-assisted lymphovenous anastomosis.

**Results:**

A total of four complete robotic-assisted lymphovenous anastomoses were performed in the lower legs of two patients. All anastomoses showed patency in the intraoperative indocyanine green imaging, demonstrating the feasibility of total robotic-assisted lymphovenous anastomosis.

**Conclusion:**

Total robotic-assisted lymphovenous anastomosis is a safe and feasible surgical technique. Microsurgical robotic assistance enhances the surgeon’s precision and improves the ergonomics, thus ameliorating surgical endurance. Further studies are required to compare this new technique to a traditional manual approach regarding the surgical outcome and cost efficiency. Future technical refinements such as a greater variety of microsurgical instruments and the possibility of automated robotic surgery could further expand the application of robotic assistance in lymphatic surgery.

**Supplementary Information:**

The online version contains supplementary material available at https://doi.org/10.1007/s11548-026-03589-0.

## Introduction

Lymphedema is characterized by the pathological accumulation of protein-rich fluid in the interstitial compartment and can be divided into primary (congenital) and secondary (acquired) [1]. In high-income countries, the latter is much more frequent than the primary impairment of lymphatic drainage and typically occurs after cancer treatment, trauma, or infection [2]. Lymphedema significantly reduces the quality of life by causing a variety of physical and psychological symptoms [3, 4]. Treatment options range from conservative methods such as manual lymphatic drainage and compression therapy to microsurgical reconstructive techniques, where lymphatic drainage is restored [5, 6]. The surgical management of lymphedema was rapidly expanded over the last few decades with a growing body of literature describing different supermicrosurgical interventions such as lymphovenous anastomosis (LVA), vascularized lymph node transfer (VLNT), and lymph node-to-vein anastomosis (LNVA) in the peripheral and central lymphatic system [7–10].

In the last decade, two microsurgical robotic platforms, namely the Symani® Surgical System (Medical Microinstruments MMI®, Jacksonville, FL, USA) and the MUSA (MicroSure, Eindhoven, The Netherlands), have been introduced into lymphatic surgery and received the certification for clinical application in Europe (CE mark). The first cases of robotic-assisted LVA were published in 2020 using the MUSA robot and in 2021 using the Symani® Surgical System [11, 12]. Ever since, an increasing number of studies described the use of these microsurgical robotic platforms for the reconstruction of the peripheral and central lymphatic system [13–15]. These systems offer different benefits such as enhanced precision, improved ergonomics, and the possibility of telemicrosurgery [16]. So far, microsurgical robotic platforms have exclusively been used for the anastomosis of lymph and blood vessels as well as for nerve coaptation [17]. The performance of a LVA presents one of the most delicate steps in lymphatic reconstruction, thus requiring a maximum of precision. However, the tissue handling during the dissection of lymph vessels presents another crucial step demanding an equal amount of precision to avoid damage on the fragile lymphatic structures. To date, only manual lymph vessel dissection has been described in the literature.

This study presents, to the best of our knowledge, the first total robotic-assisted LVA worldwide, performing both the vessel dissection and the lymphatic bypass with the Symani® Surgical System. To illustrate this new technique, we describe its application in two different patients presenting with lower extremity lymphedema. Further, we discuss the implications of this newly achieved milestone for microsurgical robotics by putting it into the context of current literature on microsurgical robotic assistance in lymphatic surgery. Finally, this study aims to elaborate technical refinements required in future generations of current microsurgical robotic platforms to push the limits of robotic assistance in lymphatic surgery even further.

## Methods and materials

Using the Symani® Surgical System, total robotic-assisted LVAs were performed in two patients presenting with lower extremity lymphedema. Informed consent for the publication of clinical data and images was obtained from both patients.

### Patient characteristics and perioperative lymphedema assessment

The first patient, a 32-year-old female, presented with bilateral secondary lower extremity lymphedema stage 2 ISL (International Society of Lymphology) following childhood burn trauma with a long history of subsequent reconstructive interventions during adulthood. The patient received conservative treatment such as manual lymphatic drainage and compression garments for several months; however, no sufficient alleviation of the symptoms could be achieved. Due to deformities resulting from burn scars, circumference measurements and volume calculations were performed only for the non-deformed parts of the legs. Thus, the volume of the left leg was calculated using the sum of 10 circumference measurements and of the right leg using the sum of 16 circumference measurements of regions where the legs were not affected by burn scars.

The second case involved a 27-year-old female suffering from primary lymphedema stage 2 in the left leg for four years as well as lipedema stage 2 ISL in both upper legs. At the time of presentation, the patient had already received manual lymphatic drainage and compression garments, which she chose to discontinue due to the absent improvement of the lymphedema. One week before the surgery, daily manual lymphatic drainage and additional bandaging were resumed. After the surgery, compression therapy was continued immediately and manual lymphatic drainage was resumed after 2 and 3 weeks, respectively.

As part of the routine preoperative diagnostics at our department, all patients presenting with upper or lower extremity lymphedema receive indocyanine green (ICG) lymphangiography as well as high-frequency ultrasound to identify suitable lymph collectors and venous vessels for LVA. Further, in each case, extremity volume is objectively assessed by perometry and circumference measurements as well as by photographic documentation. Postoperatively, these examinations are repeated at standardized intervals.

### Total robotic-assisted LVA: set-up and surgical technique

The Symani® Surgical System was used in combination with an optical microscope (KINEVO® 900; Carl Zeiss AG, Oberkochen, Germany) for maximum depth perception and resolution of the lymphatic vasculature. Both procedures were performed by the senior author. The surgeon (NL) operated the robotic platform remotely by using two freely movable controllers and foot pedals. Both the controllers and the operating chair were draped with sterile covers allowing the surgeon to quickly shift between the surgical site and the remote operator site. A technical assistant was positioned sterile next to the operating field to support the surgeon and change the instruments at the tips of the robotic arms required for different surgical steps. Both surgeries were performed with the patient in a supine position and under general anesthesia.

**Fig. 1 A:**
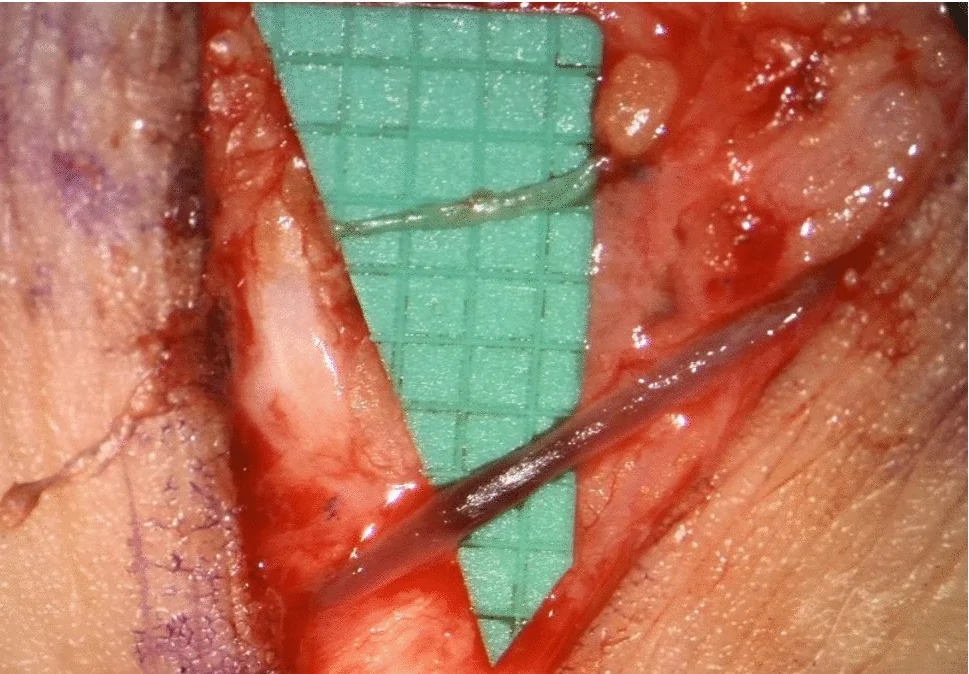
0.3-mm lymph vessel (top) and 0.5-mm vein (bottom) after dissection with microforceps and microscissors

**Fig. 2 A:**
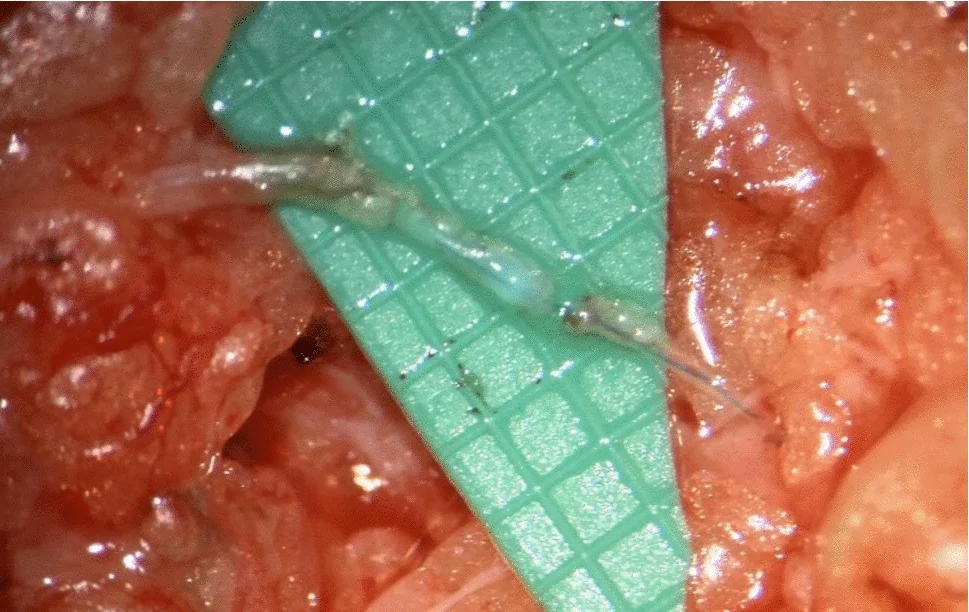
0.3-mm lymph vessel (left) and 0.5-mm subcutaneous vein (right) after robotic-assisted microdissection and intravascular stent placement (7–0 nylon)

**Fig. 3:**
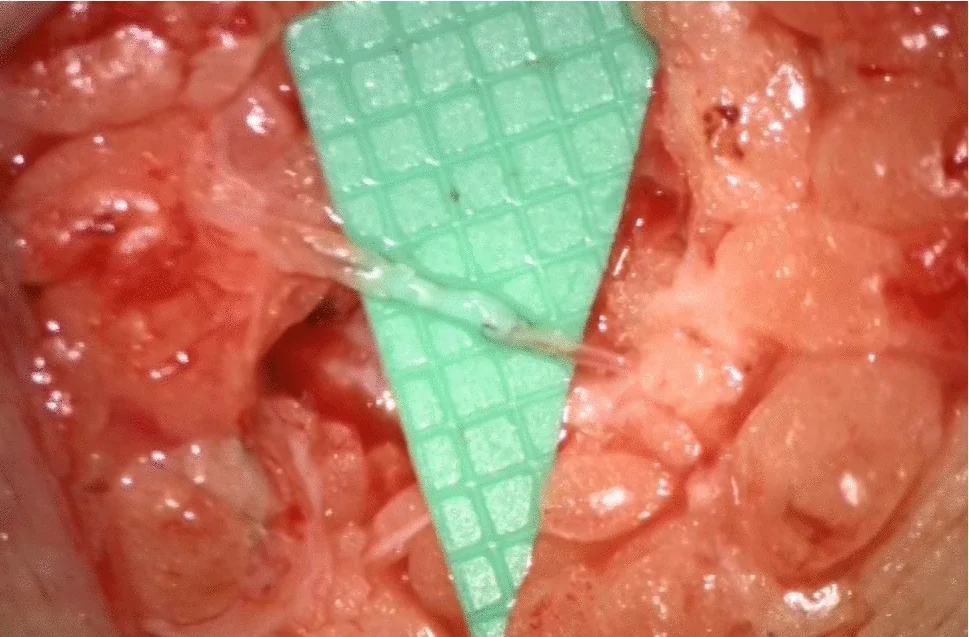
Patent robotic-assisted anastomosis (0.3 mm) using 11–0 nylon suture

**Fig. 4:**
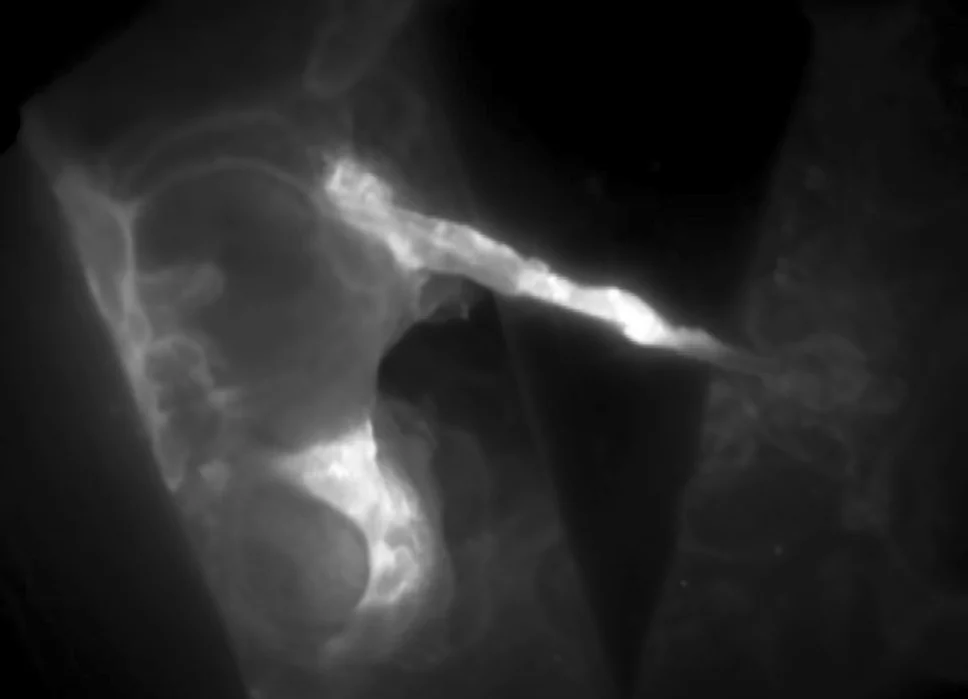
ICG-fluorescence showing a patent anastomosis

As an initial step, a combined mixture of ICG and patent blue dye was applied into the interdigital spaces of the foot as well as to the medial and lateral ankle to visualize lymph collectors using a fluoroscope (Fluobeam, Fluoptics Imaging Inc. Mass.). Subsequently, the incision sites were performed on both sides on the dorsum of the foot and the distal lower leg, and the decision to use the robotic system on which leg was made randomly in patient 1. Afterward, the Symani® Surgical System was moved into position and the surgeon switched to the chair with the freely movable robotic controllers. Using the microsurgical robotic platform equipped with a 3.5-mm microforceps and 3.5-mm microscissors, the lymph collectors and nearby veins were carefully dissected (Figs. 1 and 2). Video recordings of robotic-assisted vessel dissection are provided in Online Resource 1 and Online Resource 2. Following this step, the microdissection instruments were exchanged for a 3.0-mm supermicro needle holder with integrated suture-cut function and a 3.0-mm supermicro dilator. Robotic-assisted end-to-end LVAs were performed on lymph vessels measuring less than 0.5 mm in diameter using an 11–0 nylon suture (Online Resource 3 and Online Resource 4). The integrated suture-cut function of the supermicro needle holder is demonstrated in Online Resource 5. Performance of deep vessel dissection was perfomed in a total robotic fashion as well (Online Resource 6). Intraoperative photo documentation of a completed total robotic-assisted lymphovenous anastomosis is shown in Fig. 3. Patency of the anastomoses was checked with ICG-imaging (Fig. 4). For wound closure, the surgeon returned to the surgical site to place manual subcutaneous sutures with PDS 5–0. Finally, additional liposuction was performed in the affected extremities and compression bandaging was applied with cotton padding and elastic bandages.

## Results

Total robotic-assisted LVAs including both robotic-assisted lymph vessel dissection and anastomosis were performed, introducing a novel technique in robotic-assisted supermicrosurgery.

A total of four complete robotic-assisted LVAs were performed in the lower legs of two patients. The first patient received two total robotic-assisted LVAs on the left lower leg and two manual LVAs on the right leg. Both legs received additional liposuction of 900–1000 ml. The second patient received two total robotic-assisted LVAs in the left lower leg.

All anastomoses showed patency in the intraoperative ICG imaging. No intra or postoperative complications occurred. In both cases, the surgeries were conducted recently; therefore, long-term follow-ups for the outcome assessment are still pending. However, no differences in surgical quality or clinical course between the manual and total robotic-assisted techniques were observed during the procedure or within the 1st weeks postoperatively.

The short-term effect of total robotic-assisted LVA was quantified using pre and postoperative circumference measurements and volume calculations. In patient 1, both legs showed a decrease in volume in the 2-month follow-up (Table 1). Comparing the preoperative volume with the 2-month follow-up, a relative volume decrease of 14.89% in the left leg with total robotic-assisted LVA and 5.9% in the right leg with manual LVA was noted. However, based on the small sample size, only limited conclusions can be drawn from this regarding a potential superiority of total robotic-assisted LVA. In patient 2, an increase in volume was noted in both the operated and the not operated leg in the beginning, possibly due to the additionally existing lipedema. However, during the 3-month follow-up, the swelling decreased noticeably and the patient reported a softening of the tissue and reduced heaviness of the leg.

**Table 1:** Preoperative and postoperative volumes calculated from the sum of 10 circumference measurements of the left leg and 16 circumference measurements of the right leg. The values in parentheses indicate the relative volume change compared with the preoperative volume

	*Volume (ml), tRA-LVA*	*Volume (ml), mLVA*
*Preoperative*	4905.2	9466.7
*2-week follow-up*	4538.6 (− 7.47%)	9942.5 (+ 5.03%)
*5-week follow-up*	4253.7 (− 13.28%)	9083.5 (− 4.08%)
*2-month follow-up*	4174.9 (− 14.89%)	8908.5 (− 5.9%)

*tRA-LVA* total robotic-assisted lymphovenous anastomosis, *mLVA* manual lymphovenous anastomosis

Having a long-standing experience in robotic-assisted LVA, the surgeon reported no difficulties during the performance of robotic-assisted vessel dissection. In fact, the increased precision provided by the robotic platform offered a major benefit for the handling of the delicate tissue with vessel diameters measuring less than 1 mm. Further, the surgeon noted improved ergonomics through the use of a chair with armrests and an increased range of motion provided by the wristed microinstruments, which eliminated the need to operate at inconvenient angles and thereby increased the surgeon’s endurance. The exchange of the dissecting and suturing instruments was quick amounting to about 60 s.

## Discussion

To the best of our knowledge, we herein report on the first total robotic-assisted LVA worldwide, performing both vessel dissection and anastomosis with robotic assistance. While the feasibility and the benefits of robotic assistance for microsurgical anastomoses have been shown in multiple studies, no other steps than anastomosis of vessels and nerves have been performed with microsurgical robotic platforms so far [13, 17, 18]. By showing the feasibility and safety of robotic-assisted vessel dissection, we broadened the previous application area of microsurgical robotic platforms.

The patency of all anastomoses was shown intraoperatively with ICG imaging, proving the technical feasibility of total robotic-assisted LVA. Considering the small case number and the short follow-up period, no conclusion regarding the efficacy of this new technique can be made at this point in time. However, when comparing total robotic-assisted LVA with hand-sewn anastomosis within the same patient, total robotic-assisted appeared to reduce extremity volume more over a 2-month follow-up.

The Symani® Surgical System enhances precision through 7–20× motion scaling and tremor filtration, leading to a reduced risk of vessel damage and to less tissue injury during the vessel dissection and anastomosis. Thus, in our cases, we were able to dissect and anastomose fragile vessels measuring 0.2 to 0.5 mm in diameter without any difficulty. Further, Chen et al. found that with the improved precision provided by the Symani® Surgical System, anastomosing a 0.8-mm vessel versus a 0.1-mm vessel is perceived as almost equal regarding technical difficulty [15]. Moreover, enhanced precision could allow less experienced surgeons to perform microsurgical procedures, potentially increasing the number of surgeons offering technically demanding interventions on the peripheral and central lymphatic system in the future. Wessel et al. found that especially surgical novices showed a steeper learning curve in robotic-assisted microsurgery than in manually performed vessel anastomosis. Further, the study found that the surgical quality of the final anastomoses performed during a one-time training session was significantly higher in the robotic-assisted anastomoses compared to those conducted manually [19]. However, prior to operating with a microsurgical robot on patients, surgeons of all levels of experience must complete several training sessions to get used to the handling of the robotic system [20].

Combining the Symani® Surgical System with a 3D exoscope, the need for the surgeon to sit in close proximity to the patient and put the hands on the patient is eliminated. Therefore, movements of the hands do not result in corresponding shaking of the operating field. Further, the teleoperated platform allows the surgeon to sit on an ergonomical chair, which leads to improved posture, thus supporting longer surgical endurance as well as decreasing the risk of musculoskeletal disorders in the long term, whereby the latter poses a concerning issue for microsurgeons [21, 22]. Moreover, telemicrosurgery could allow the remote performance of selected critical steps of interventions or even whole interventions from a distant geographical site in the future, leading to easier access to specialized treatment of lymphedema. The feasibility of telesurgery has already been shown in other specialties with non-microsurgical robotic platforms [23, 24].

In the robotic set-up, the microinstruments are mounted on thin shafts measuring 12 to 15 cm in length [25]. This facilitates surgical access to deeper anatomical regions such as the deep neck lymphatics, intraoral structures, and the axilla, which can be difficult to reach with a manual approach. Recently, the deep neck lymphatics have gained increased attention following the discovery of the brain’s lymphatic system and the understanding of the important role of deep cervical lymph nodes in the lymphatic drainage of the central nervous system [26]. Current literature suggests that neurodegenerative diseases such as Alzheimer’s disease could be alleviated by improving glymphatic and meningeal lymphatic drainage through reconstructive lymphatic procedures such as deep cervical LVA [27]. Both increased precision and better access to deeper anatomical planes provided by microsurgical robotic platforms may represent a major benefit. Taking this idea one step further, a potential future goal could be to operate directly on meningeal lymphatic vessels, which would make the use of robotic assistance indispensable as the width of the vessel lumen is in the micrometer range [28, 29].

Total robotic-assisted LVAs including both vessel dissection and LVA may result in slightly longer operating times, directly resulting from the prolonged duration of the surgical steps due to the motion scaling and indirectly from the time needed for the set-up of the robot and the intraoperative switching between manual and robotic-assisted techniques. However, in recent studies, a decrease of operating times in robotic-assisted anastomoses was observed with growing experience [11, 19, 30], which is why the duration of total robotic-assisted LVA can be expected to approach the duration of manual LVA over time as well.

Increased costs caused by robotic platforms are repeatedly discussed in the literature [14, 17, 31]. Higher precision provided by the Symani® Surgical System during all microsurgical steps including vessel dissection may reduce tissue damage, potentially offering a medical benefit and justifying increased costs. Furthermore, 13 European microsurgery centers concluded that using robotic assistance in microsurgery may reduce invasiveness and patient morbidity, thus conceivably lowering costs by avoiding complications, which may offset or even outweigh the expenses of the robotic platforms [16]. Further, Hughes et al. noted that arising costs of using robotic assistance are volume-dependent, thus costs per procedure decrease with an increase in procedure volume [32].

Future versions of the prototype of the Symani® Surgical System may address current technical drawbacks such as the fixed angles of the robotic arms, which increase the shaft-to-shaft distance at the skin level when the arms are lowered, potentially necessitating longer incisions for interventions on deep-lying structures [33]. Therefore, the development of flexible arms could allow an even less invasive approach when performing LVAs in deeper regions. By not only adapting the robotic arms but also the variety of available instruments, microsurgical robotic assistance could be expanded to further surgical steps and interventions. Thirteen European microsurgery centers concluded in a consensus paper that automation of microsurgical robotic platforms is the top long-term goal of robotic-assisted microsurgery [16]. Similar to the possibility of telemicrosurgery, the introduction of robotic automation would allow less experienced microsurgeons to perform highly demanding microsurgical procedures, as difficult steps could be taken over completely by the robotic platform.

Limitations of this study included the small number of patients and the short follow-up period as the interventions have been performed recently. Therefore, a conclusion regarding efficacy and a comparison to manual LVAs regarding the outcome was not possible. Despite the limitations, this study introduced a new technique of robotic-assisted lymphatic surgery by demonstrating the feasibility of total robotic-assisted LVAs including the execution of the vessel dissection. Hereby, the potential of microsurgical robotic platforms was further exploited, providing improved precision and ergonomics not only during vessel anastomosis but throughout all microsurgical steps during this procedure. Lastly, both the benefits and current challenges of microsurgical robotic platforms were highlighted, thus outlining future directions for both the manufacturers and the users of these systems. Further studies with larger patient cohorts and longer follow-up periods will allow the assessment of the efficacy of total robotic-assisted LVAs, and future technical refinements may support the continued expansion of microsurgical robotic platforms.

## Conclusion

Robotic assistance is a powerful tool in supermicrosurgery and is especially useful in lymphatic reconstruction by enhancing the surgeon’s precision, range of motion, and ergonomics. In a small patient cohort, this study demonstrates the safety and feasibility of using a dedicated microsurgical robotic platform not only for the vessel anastomosis but also for lymph vessel and vein dissection, marking an additional expansion of previous applications of robotic assistance in microsurgery. Further research regarding the efficacy of this new technique should be conducted with larger patient cohorts and longer follow-up periods. Additionally, a direct comparison of robotic-assisted and manual vessel dissection and anastomosis regarding surgery time, outcome, and costs should be performed. Future refinements of current prototypes may broaden and consolidate the current scope of application of microsurgical robotic systems and may facilitate access to highly specialized lymphatic microsurgery.

## Supplementary Information

Below is the link to the electronic supplementary material.

Supplementary file1 (MP4 52690 KB)

Supplementary file2 (MP4 48199 KB)

Supplementary file3 (MP4 122167 KB)

Supplementary file4 (MP4 82354 KB)

Supplementary file5 (MP4 29750 KB)

Supplementary file6 (MP4 50956 KB)

## Data Availability

Research data are not shared.
